# Midwives’ knowledge and perspectives on neonatal resuscitation and survival before and after Helping Babies Breathe training: a qualitative study in Uganda

**DOI:** 10.1136/bmjopen-2024-094012

**Published:** 2025-02-07

**Authors:** Susanna Myrnerts Höök, Marielle Abrahamsson, Sarah Namusoko, Josaphat Byamugisha, Anna Bergström

**Affiliations:** 1 Department of Global Public Health, Karolinska Institutet, Stockholm, Sweden; 2 Department of Global Public Health and Primary Care, University of Bergen, Bergen, Norway; 3 Sachs Children and Youth Hospital, Södersjukhuset AB, Stockholm, Stockholm County, Sweden; 4 Department of Health Policy, Planning and Management, School of Public Health, Makerere University, Kampala, Central Region, Uganda; 5 Department of Obstetrics and Gynaecology, School of Medicine, College of Health Sciences, Makerere University, Kampala, Uganda; 6 Department of Women’s and Children’s Health; Centre for Health and Sustainability, Uppsala Universitet, Uppsala, Sweden

**Keywords:** NEONATOLOGY, Cardiopulmonary Resuscitation, MEDICAL EDUCATION & TRAINING

## Abstract

**Abstract:**

**Objectives:**

Birth asphyxia is a significant factor contributing to neonatal mortality, particularly in low- and middle-income countries where most neonatal deaths occur. Encouraging women to deliver in hospitals has become a pivotal strategy. Numerous training programmes, such as Helping Babies Breathe (HBB), have been designed to impart neonatal resuscitation and infant care skills to support breathing at birth. Limited attention has been given to exploring the perspectives and experiences of midwives and their hospital managers in translating the acquired knowledge from these programmes into practice. This study aims to explores the understanding, perspectives, and first-hand experiences related to the factors impacting neonatal resuscitation practices and survival, both pre-HBB and post-HBB training.

**Design:**

Qualitative individual interviews and focus group discussions study. A data-driven inductive content analysis approach was used for the analysis.

**Setting:**

The high-risk labour ward and theatre at a National Referral Hospital, Uganda.

**Participants:**

45 clinically active midwives were enrolled; all had recently completed the HBB training programme.

**Intervention:**

Semistructured individual interviews (n=2) and focus group discussions (n=43, distributed across seven groups) were held from 26 April to 4 May 2018. Discussions were audio-recorded and transcribed verbatim.

**Results:**

Three emerging themes illustrated midwives’ knowledge, opinion on and experience of neonatal resuscitation and survival. Excessive workload, limited access to clean equipment, and ethical dilemmas hampered performance and neonatal survival. Midwives, facing inadequate support, strived to ensure patient safety. While HBB training addresses malpractices, additional training was needed.

**Conclusions:**

Midwives had few opportunities to change their workload and improve their education. This highlights the need for a closer examination of the challenges faced by healthcare providers in ensuring effective neonatal resuscitation and survival in low-resource settings. To address this, we propose better routines for organising work, cleaning and maintaining equipment, and implementing better training routines.

**STRENGTHS AND LIMITATIONS OF THIS STUDY**

- This is one of few studies to explore the work environment of a high-risk labour ward and operating theatre in a large referral hospital within a low-resource setting directly from the perspectives of its midwives.
- While certain findings may be context-specific and time-specific, our main conclusions are broadly applicable and consistent, aligning with those reported in other settings and highlighting the role of implementation science.
- The study gains strength from a substantial number of midwives and extensive material.
- The study benefits from appropriately sized and well-composed focus groups, fostering open and honest discussions.

## Introduction

 Globally, the main causes of neonatal deaths (death before 28 days of life) are preterm birth, intrapartum-related complications (birth asphyxia) and sepsis,^1^ with 96% occurring in low- and middle-income countries.^2^ Good progress has been made, such as in Uganda, where the neonatal mortality rate fell from 40 deaths per 1000 live births in 1990 to 18.4 in 2022.^3^ However, challenges persist in Uganda, including a high incidence of early neonatal deaths and stillbirths due to birth asphyxia.^1 4 5^

Effective positive pressure ventilation provides the single most important component of successful neonatal resuscitation.^6^ Despite increased skilled care attendance at birth, quality of care at the facility level remains a concern, hindering mortality rate reduction.^7 8^

In low- and middle-income settings, health personnel often lack extensive airway management experience and reliable monitoring equipment for neonatal resuscitation. Programmes like Helping Babies Breathe (HBB) have been developed to address this issue. HBB, recognised as a breakthrough innovation for infants, emphasises skilled attendance at birth and basic neonatal resuscitation skills.^9 10^ The training emphasises ventilation skills and stresses the importance of initiating ventilation using a self-inflating bag and mask within the ‘Golden Minute’ post-birth if the infant struggles to breathe.

Implemented in countries like Tanzania and Nepal, HBB has significantly reduced neonatal mortality rates.^11^,^13^ While initial training yielded commendable skills, retention of skills has been found to decline without periodic re-training, highlighting the need for ongoing support.^14^,^17^ Research on HBB advocates the need for improvements in various aspects of the training, including longer training periods.^18^

A 2017 Cochrane review of 31 qualitative studies from Africa, Asia and Latin America examined factors impacting care delivery by skilled birth attendants in low- and middle-income countries.^19^ It identified various determinants such as access to training, supervision, staff levels, workloads, salaries, living conditions and access to well-equipped healthcare facilities with essential amenities like water, electricity and transport.

Efforts directed at understanding the need among health workers to adopt new practices are indicating that strategies to promote the uptake of evidence-based practices need to be tailored to encompass the needs in the specific context. Further, most studies do not delve into the retention of knowledge and skills or the challenges health workers face in applying practices such as HBB in routine practice.^20^ Instead, most studies evaluating HBB intervention in low-income countries focus on pretraining and post-training assessments of birth attendants’ knowledge and skills.^12 16 21 22^

In Uganda, HBB is integrated into national standards,^23^ yet adherence to standard practices remains a challenge, indicating the need for further support^24^ and exploration of factors contributing to poor adherence to the HBB protocol. This study aims to understand the experiences of midwives (MWs) trained in HBB in their effort to implement HBB in daily practice, exploring factors influencing neonatal resuscitation practices and survival. It also explores the sociocultural perspectives surrounding birth asphyxia, providing insights crucial for improving outcomes.

## Materials and methods

This is a qualitative study adopting an exploratory design, gathering data through semistructured individual interviews (IIs) and focus group discussions (FDGs). The analysis was conducted using a data-driven inductive content analysis.^25^

### Setting

Uganda, with a population of approximately 45 million people, is classified as a low-income country, with roughly 30% living in poverty.^26^ This study was conducted with staff from the Department of Obstetrics and Gynecology within the Mulago National Referral and Teaching Hospital in Kampala, Uganda, where healthcare professionals assisted in about 25 000 deliveries in 2018. The semistructured IIs and FDGs were conducted in a secluded space near the hospital.

### Study population

The sampling strategy used a combination of purposive and convenience sampling. The principal investigator (SMH) is a certified master trainer of HBB and conducted HBB training for MWs at the Mulago National Referral Hospital from 29 November to 13 December 2017, as part of a randomised controlled trial.^27^ Potential participants included those who had taken part in a previous HBB study in 2016^24^ and/or had a minimum of 3 months of experience at the high-risk labour ward and obstetric theatre of Mulago National Regional Referral Hospital from 10 November 2015 to the end of 2017. SMH provided a brief 5-minute PowerPoint presentation explaining the study objective. Interested MWs provided their names and telephone numbers in writing.

In early April 2018, coauthor SN contacted all interested MWs who met the inclusion criteria through a phone call. MWs in roles such as centre or area managers were excluded, as their daily responsibilities do not involve neonatal resuscitation, and it might be challenging for MWs to express their opinions freely in the presence of higher-ranking staff. To ensure well-matched groups, participants were asked about their age, education level, neonatal resuscitation training and specific HBB training (table 1). This information was used to create groups with similar experiences, fostering a comfortable environment for open discussion. A total of 45 individuals, all women, agreed to participate. Two in-charge MWs involved in daily resuscitation activities were selected for IIs, while the other 43 MWs were organised in focus groups of 5–7 members each. Exclusion criteria encompassed nursing students, staff and students from different institutions or health facilities.

**Table 1:** Composition of individual interviews with in-charges (II) and focus group discussions (FGD) and characteristics of the participants.

(II and FGD numbers)	II 1–2	FGD 1	FGD 2	FGD 3	FGD 4	FGD 5*	FGD 6	FGD 7
Group size	2	7	5	7	6	7	5	6
Age (years)								
20–30			5	1			1	2
31–41		4		1	4	4	1	1
42–52	2	3		5	2	2	1	3
>52							2	
Highest education								
Enrolled midwife/nurse			3		1	1	1	3
Registered midwife/nurse	1	4	2	5	3	3	4	2
Nursing officer in midwifery	1	3		2	2	2		1
HBB training before NeoSupra trial†								
Yes	2	6	5	4	4	5	4	4
No		1		3	2	1	1	2
Refresher courses before NeoSupra trial†								
Yes	2	3		4	4	6	1	1
No		4	5	3	2		4	5
If No above, reason?								
Adequate knowledge								
No time								
No finances								
No opportunity		4	5	3	2		4	5
Use the HBB Action plan to guide resuscitation?								
Yes	2	7	5	5	4	5	5	6
No				2	2	1		

* Missing data on one midwife’s age and training.

† Parallel RCT on supraglottic airway in neonatal resuscitation.

### Study procedure

Written informed consent was obtained on-site following a brief verbal introduction to the study’s purpose. The IIs and FDGs were facilitated using a semistructured guide (online supplemental file 1). IIs typically lasted approximately 60 min, while FDGs spanned 90–120 min, divided into two segments, including a 10 min break. The initial part involved a discussion guided by the semistructured guide. The second part was conducted using video-elicitation methods^28^ whereby short, 2 min video clips of actual neonatal resuscitations from a previous project^24^ were viewed. To ensure anonymity, the videos were muted and edited to remove any details that could identify the resuscitator. Following each clip, the moderator used the video to elicit reflection on the scenarios and by using questions from the semistructured guide.

### Data collection and analysis

The semistructured interview guide was developed through discussions within the study team. The guide underwent a pilot phase in the initial II and the first FDG. No alterations were made following the initial testing. Data collection was conducted in English, audio-recorded, with moderation performed by SN, a social scientist with experience in moderating qualitative interviews, conducted the sessions. SMH was consistently present in a supportive capacity, taking notes and formulating follow-up questions. SN could effectively understand and translate into the local language, Luganda, when required for short clarifications of the questions. The number of FDGs was determined by saturation, signifying no addition of essential new information.^29^ The 22 hours of audio-recorded material were transcribed by SN verbatim and de-identified during the transcription process.

Content analysis, following the approach outlined by Graneheim and Lundman,^25 29^ was applied for data analysis using NVivo (QSR International). The analysis commenced with coauthors MA and SMH identifying ‘meaning units’ within the text. Meaning units involved isolating text from context and condensing them into concise items while retaining the original meaning, that is, codes. These codes underwent multiple rounds of merging and matching by coauthors MA, SMH and AB. This process continued until no new meanings emerged. Each code was systematically re-evaluated and compared with others for enhanced trustworthiness. The data from both IIs and FDGs were primarily analysed separately, identifying separate subcategories. When needed, the subcategories were revisited and rephrased. In such cases, the data set was reopened and reanalysed to sustain a comprehensive analysis. The last step included merging IIs and FDGs data before identifying the categories to guarantee anonymity for all participants.

MA and SMH subsequently established categories, subthemes and overarching themes. These were discussed with AB, SN and JB to reach a consensus on the structure and conceptualisation. The final themes were carefully named, and the findings were articulated using a blend of narrative text and excerpts from the data sets, ensuring a comprehensive demonstration of each theme and reinforcing validity.^30 31^ The manuscript was written adhering to the Standards for Reporting Qualitative Research checklist.^32^

### Confidentiality

The recordings were securely stored in a locked drawer before transcription, and access was restricted to the co-investigators of this study. The recordings will be permanently deleted after publication. Participant identities were kept confidential and anonymous to individuals not involved in the study. Citations from participants were anonymised. The results from IIs and FDGs were combined to prevent the identification of specific staff members.

### Patient and public involvement statement

Patients or members of the public were not involved in this study.

## Results

**Figure 1:**
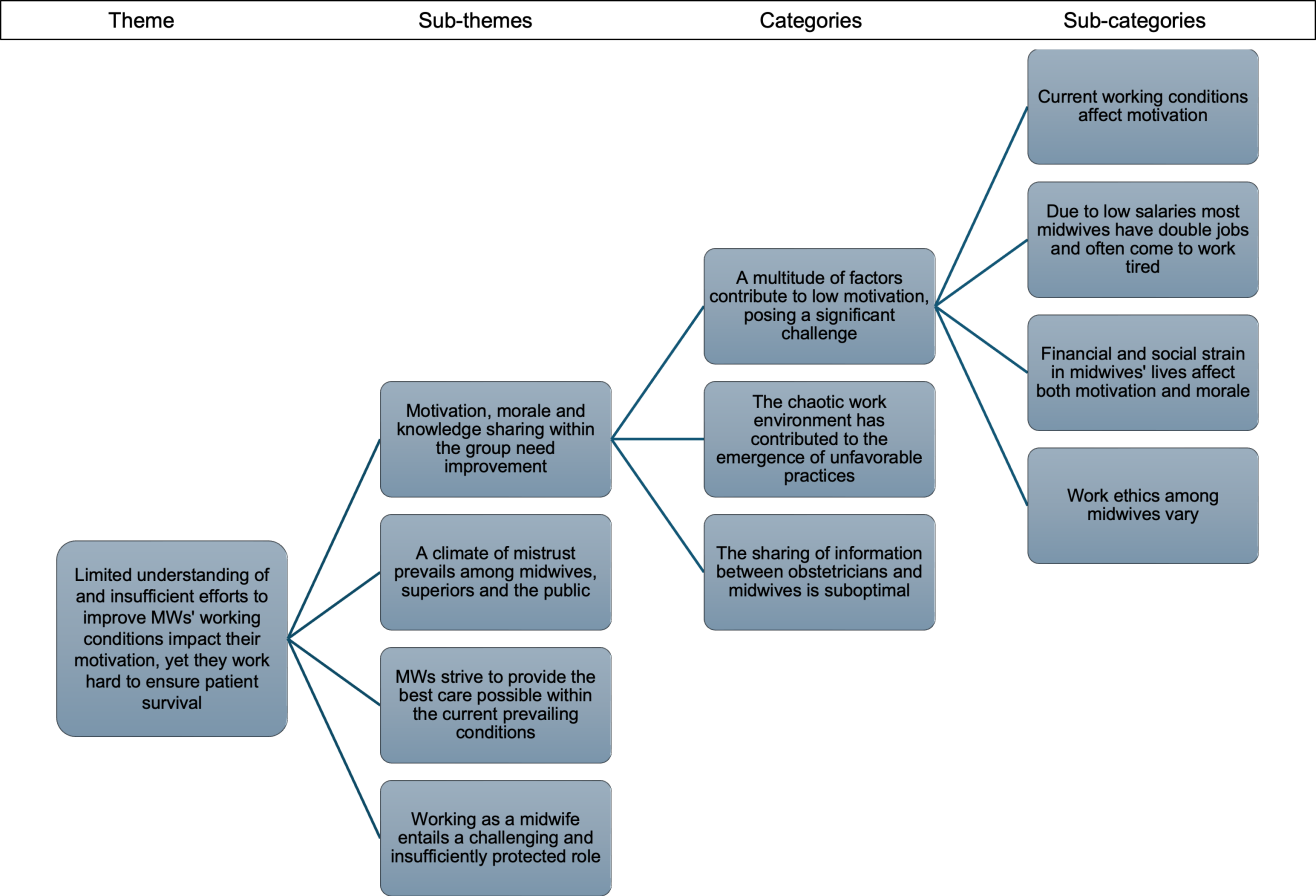
Description of the development of one of the themes through subcategories, categories and subthemes. MWs, midwives.

The results illustrate how MWs trained in HBB experience their effort to resuscitate neonates in daily practice and the sociocultural perspectives surrounding birth asphyxia, providing insights for improving outcomes. The data analyses resulted in three themes, presented below alongside summaries of their specific subthemes with illustrative quotes. A detailed description of the 23 categories and the nine subthemes forming the three themes is found in table 2. A detailed description of the development of one of the themes through subcategories, categories and subthemes is presented in figure 1.

**Table 2:** Presentation of the themes, subthemes and categories

Themes	Subthemes	Categories
Enormous workload, insufficient access to clean equipment and ethical dilemmas negatively affects both performance and NR	Establishing effective routines and allocating improved resources is crucial to achieving elevated hygienic standards	Improving handling of equipment is crucial
		Maintaining high hygiene standards is impossible, leading to high risk of cross-infections
	High patient volume and inadequate supplies adversely affect MWs' performance, impacting neonatal survival	Enhancing the work environment and ensuring adequate equipment is essential
		Meeting the golden minute is unattainable given the existing conditions
		Patients die every day due to lack of equipment
		The overwhelming numbers of patients negatively impacts every aspect of NR
	MWs face challenging ethical decisions due to extremely demanding workload and are influenced by cultural beliefs	Cultural believes regarding neonatal care impact both patients and MWs
		In the absence of proper equipment MWs find their own way of assessing the situation
		MWs daily make difficult ethical decisions involving two patients: the mother and the child
Limited understanding of and insufficient efforts to improve MWs' working conditions impact their motivation, yet they work hard to ensure patient survival	A climate of mistrust prevails among MWs, superiors and the public	The feeling of mistrust exists both among MWs and towards their superiors
		The management's lack of understanding regarding the unique nature of perinatal work negatively impacts both patients and work environment
	MWs strive to provide the best care possible within the current prevailing conditions	MWs possess a strong determination to ensure the survival of their patients
		Working as a MWs creates strong emotions and will to cooperate
	Motivation, morale, and knowledge sharing within the group need improvement	A multitude of factors contribute to low motivation, posing a significant challenge
		The chaotic work environment has contributed to the emergence of unfavourable practices
	Working as a MW entails a challenging and insufficiently protected role	MWs encounter many situations where they are exposed to dangers with limited support from superior
		MWs predominantly work without support
While HBB training successfully identified and addressed malpractices, there is a need for additional knowledge and skills in neonatal resuscitation	Education is said to be promoted at the hospital, yet doctors and MWs education need improvement for best practice	Despite having HBB training, there is a knowledge gap concerning perinatal care among MWs
		It is crucial that all personnel, including doctors, undergo HBB training
	HBB training revealed existing malpractices and fostered improvements in newborn care practices	While both internal and external education are promoted, there's substantial room for improvement
		HBB has improved every aspect of NR
		MWs now perform continuous NR until certain the baby is deceased
		Prior to HBB, numerous malpractices were commonly employed by everyone

HBB, Helping Babies Breathe; MWs, Midwives; NR, Neonatal Resuscitation

### Theme one: enormous workload, insufficient access to clean equipment, and ethical dilemmas negatively affect both performance and neonatal resuscitation

Establishing effective routines and strategically allocating enhanced resources are fundamental steps towards attaining elevated hygienic standards in healthcare settings.

*Now, like the autoclave, it can be down like for three months. Now, like at the moment, we have been having the autoclave on level three working, the autoclave of family planning is down, the one of the theatre went down. They brought another one, but it is also not so good. It took like four months to bring that one, and the whole hospital is now having one working autoclave.[Participant 6, FDG 1]*

The confluence of high patient volumes and inadequate supplies poses significant challenges to the performance of MWs, thereby directly impacting neonatal resuscitation and neonatal survival rates.

*We are prepared, but things are not enough. So, when you reach there, you say my God! Where is the ambubag? Or you find another midwife resuscitating a baby, and you are stuck. You have only two working ambubags. Or you find two babies being resuscitated, and you have brought another one. You have to stand and wait. Now yours is improving, give me, and I put this ambubag to resuscitate.[Participant 4, FDG 1]*

MWs also highlight an inadequate referral system leading to delays in arrival at the hospital. When facing these challenges, MWs often encounter complex ethical dilemmas that are further complicated by the relentless demands of their workload and the subtle yet significant influence of cultural beliefs. For example, the MW is often the sole caregiver responsible for and available to both the newborn and the mother in labour. If both experience complications simultaneously, the MW must make the critical decision of whom to prioritise first. This often occurs while the MW is simultaneously responsible for several other women in active labour, all of whom may need her attention.

*When you get a baby who is asphyxiated, that’s when it’s a challenge. You leave the one who has delivered, remove another glove and run with the baby for resuscitation. The other mother is remaining there, and remember is going to push. So, when you are there on the resuscitation,“Eh, musawo (midwife) come, come, am doing badly”. You leave the baby and run to the other one to remove the baby. So, you find this baby, if you don’t find a person and tell them to help you with the bagging to ventilate, then you will lose that baby in the resuscitation area, and also, if you don’t help the other mother who is pushing, you will also get problems.[Participant 4, FDG 4]*

In addition, cultural beliefs often emphasise practices such as suctioning or shaking the newborn upside down. For example, the mother or attendant in-laws may insist that the MW perform these actions. However, the MW is trained to abstain from such practices, as they can delay appropriate resuscitation measures. Despite this, these practices are sometimes perceived as essential for the baby’s survival or future health, potentially leading to conflicts or blame directed at the MW. This means that MWs are frequently confronted with difficult decisions that are influenced by both professional obligations and cultural perspectives, all while managing their demanding workloads.

*Even the mothers still want that practice because they say that their babies will swallow the secretions if not tilted (‘ocumira’). Most of those mothers come with in-laws or parents as attendants who believe that if the baby is not tilted, it will aspirate and get breathing problems in the future.[Participant 2, FDG 5]*

### Theme two: limited understanding of and insufficient efforts to improve midwives’ working conditions impact their motivation, yet they work hard to ensure patient survival

Within the realm of midwifery, there exists a pervasive atmosphere of mistrust among MWs themselves, their superiors, and the broader public. There is a widespread tendency to blame individual MWs when complications arise rather than considering these incidents within the context of the suboptimal working conditions described. Additionally, MWs report being threatened by in-laws or friends of mothers giving birth. In some cases, MWs have faced legal action, accused of malpractice or even of stealing babies from the hospital. This creates a work environment where MWs feel unsafe and, at times, hesitate to assist when help is needed, fearing accusations of wrongdoing.

*The whole night you work, you deliver 20 mothers, in the morning, the immediate supervisor will only look for mistakes. First, say thank you. But when you first look for mistakes? How many mothers died in the night? If you have lost one after a month, they will call everybody: “Sue that one and take her to court!”. There is even nobody on your side. How many mothers? 100. How many MWs were on duty? There were three. How many babies were delivered in 24 hours? 120 babies. How many babies died? One. You don’t even ask how did the baby die. They just call the police officer. Let her go in.[Participant 7, FDG 4]*

Due to limited access to supplies at the hospital, patients are required to provide supplies and medicines themselves or bring money for the MWs to procure them. However, this is not always widely known, as authorities often withhold this information from the public. As a result, there is a widespread perception that the MWs keep the money for themselves.

*If the media is telling people that the mama kits are there and the drugs are there, everything is there, and if those MWs are asking for money, report them. And we have name tags, she will just write your name down, and tomorrow you will appear in the news on TV.[Participant 6, FDG 5]*

Despite having to struggle with these prevailing conditions, most MWs remain steadfastly dedicated to providing optimal care to their patients.

*Many MWs have learnt to use their hands without needle holders. So that’s the challenge we have, and with this HIV, you have to be very careful and conversant of doing without a needle holder. It’s a challenge, but we improvise. That’s how we go on.[Participant 7, FDG 3]*

Nevertheless, there is a pressing need to bolster motivation, uplift morale and foster a culture of robust knowledge sharing within the midwifery community. The profession of midwifery is inherently physically and mentally demanding, yet often lacks the requisite safeguards and protections necessary to ensure the well-being of its practitioners.

*You go back to upgrade and get your degree but get to the time of retirement when you are still getting the salary of an enrolled midwife. That is very demotivating. You have invested, but you are not getting anything out of it.[Participant 4, FDG 6]*

*At least they should be on our side. Mistakes are to humans, maybe we do make them sometimes, but we should count on our bosses. We should work together with them, but we are not recognised at all.[Participant 4, FDG 5]*

### Theme three: while HBB training successfully identified and addressed malpractices, there is a need for additional knowledge and skills in neonatal resuscitation

Education is actively promoted within the hospital environment; nevertheless, there exists a pressing need to elevate the quality of neonatal resuscitation education offered to both doctors and MWs to ensure the adoption of best practices.

*As if you find yourself, you are the person resuscitating, you have to do something else, and the person who would assist you doesn’t know what to do. But otherwise, we would have the knowledge and practice after HBB, but the person to assist is the problem. So, we have to train others so that we all have the knowledge and practice it the way we are supposed to do.[Participant 3, FDG 1]*

The introduction of HBB training has shed light on widespread malpractices and has catalysed the implementation of improvements in newborn care protocols and practices.

*Yes, because during the training, we train on dummies, and we go back to the ward and practice, and you can see that previously a baby of score four could not survive, but now, due to the skills and knowledge from HBB, a baby of score two is surviving.[II nr 2]*

## Discussion

This study has broadened our understanding of MWs’ perspectives on and knowledge about the HBB training programme, shedding light on the challenges and deficiencies in current training methods. MWs acknowledge that there exists a mistrust between colleagues and superiors. Furthermore, they express that when the management has a poor understanding of the special needs of perinatal work, it may harm patients and have a severely negative impact on the working environment. They highlighted inadequate staffing leading to heightened workloads, posing challenges in delivering optimal care. Still, MWs were committed to survival and teamwork but faced low motivation, chaotic conditions and limited support. MWs express that gaps in perinatal care knowledge persist despite HBB training and that there is a need for recurrent HBB training. However, HBB has enhanced neonatal resuscitation and reduced malpractices.

Birth asphyxia is a major contributor to neonatal mortality, placing a significant burden on already vulnerable healthcare systems. While HBB is a promising method that could save many lives, this study highlights the challenges of its implementation and underscores the need for planning efforts to address the needs of health workers adopting these best practices. Supporting this statement, a meta-analysis published in 2024 showed that healthcare workers in Eastern Africa lacked sufficient knowledge of neonatal resuscitation.^33^ Implementation science frameworks, such as the Consolidated Framework for Implementation Research (CFIR),^34^ offer a lens through which implementers can identify areas needing attention, including the characteristics of the implemented method, the roles and attributes of the individuals targeted by the innovation, and the setting of the implementation. CFIR includes the implementation process—i.e., the strategies adopted to achieve change—as a domain that can be altered based on other factors when used prospectively, or it can provide additional insight when investigating the implementation of evidence-based methods. In the current study, challenges were identified across all of the domains of the CFIR^34^ and were not isolated to the implementation of HBB. As the hospital is a referral hospital, challenges relating to the context include inadequate referral systems contributing to delays in arriving at the facility but also the need for improved conditions in which the MWs and in-charges live and operate in general. Another finding relating to context and how the working culture impedes motivation was the lack of recognition for the work which was undertaken. Further, participants described a lack of psychological and physical safety, where the threats ranged from the risk of infection due to lack of protection to being taken to court for mistakes made while working.^35^ Poor teamwork, a lack of trust, and collaboration were factors seen to negatively impact the quality of the care the MWs were able to give their patients.

Participants emphasised that implementation strategies aiming to increase motivation, such as recognition of their efforts and the provision of incentives, are important to consider for practice change. The opportunity in terms of having the required equipment and established means of maintaining them was brought forward as an essential need requiring attention for HBB to be delivered as intended. Similar findings and their implications on the delivery of service have been described previously.^20^ Structured HBB training for improved knowledge and skills is well documented.^12 16 21 22^ Participants in the current study did describe that HBB training had increased their knowledge and skills. However, MWs emphasised that training initiatives should be carefully developed to address not only how and when resuscitation should be performed but also the physiological reason behind it. A crucial example that can jeopardise the fidelity to the method is that health workers need to understand the importance of quick, uninterrupted ventilation. They also need to understand the complicated role of mucus and secretions in neonatal resuscitation, where the ABCDE approach, learnt in school, is not fully practised. The practice of suction to keep the A (airway) clear may delay the crucial practice of neonatal resuscitation, which is uninterrupted ventilation equal to B (breathing). If it is not clear that the neonate may be ventilated despite mucus, MWs may focus on going back to A to remove mucus while keeping ventilation (B), which will save the neonate. Suction will not stop until the knowledge of anatomy and the role of mucus is well understood by the health workers who should adopt HBB. The current findings add the need for more tailored approaches to ensure that strategies not only consider the capabilities of providers but also address the multitude of challenges faced by frontline health workers. Considering ways to enhance psychological safety could also contribute to overall health system strengthening and potentially increase receptiveness to future implementations.

## Methodological considerations

Our investigation was limited to a single referral hospital, focusing specifically on the high-risk labour ward and the operating theatre. Consequently, our findings are context-specific, and their transferability should be approached with caution. However, these findings provide valuable insights for potential improvements. They align with the 2017 Cochrane review by Munabi-Babigumira *et al*, which included 31 qualitative studies from Africa, Asia and Latin America^19^ examining factors impacting care delivery by skilled birth attendants in low- and middle-income countries. Previous studies also indicate that heavy workloads and insufficient preservice training in neonatal care compromise care quality and hinder optimal service delivery.^19 36 37^ It’s important to note that the study was conducted in 2018, so the findings may not fully reflect the current situation. Uganda’s mortality rate has slightly declined since the time of the study, decreasing from 20.4 in 2018 to 18.4 in 2022, according to the most recent UNICEF data.^3^ However, this rate remains significantly higher than the global target of 12 or less per 1000 live births by 2030, as set by Sustainable Development Goal 3.2. While this decline may be influenced by conditions at the study site, ongoing staff rotations, along with an increasing number of patients and referrals, could further exacerbate the mortality rate and place additional strain on MWs at the site.

Another potential limitation is the involvement of the principal investigator in the IIs and FDGs, which could influence the results. To mitigate this, a local moderator familiar with the methods was employed, and a research team conducted the data analysis. Effective qualitative research relies on participants willing to share their experiences and opinions. The study benefits from focus groups of the ideal size, typically consisting of five to seven participants.^38^ We also consider the number of IIs and FDGs to be optimal, as no additional information was revealed in the last two focus groups. The training and clinical experience of the participants, along with the composition of the groups, facilitated open and honest discussions. The principal investigator, SMH (a paediatric resident at the time), was familiar with the participants from another study at the hospital, which helped foster a comfortable environment. The moderator and author, SN, who was previously unknown to the participants, effectively guided discussions and minimised the use of medical jargon due to her qualitative experience and non-clinical background. To enhance the trustworthiness of this study, we adhered to Elo *et al*.’s^39^ checklist for content analysis throughout the research process. Key aspects of trustworthiness include credibility, dependability, transferability and conformability. Credibility was ensured through researcher self-awareness, careful phrasing of questions, and pretesting the semistructured guide. The moderator (SN), a trained social scientist, and the note taker (SM), with clinical expertise, ensured focused and relevant discussions. Dependability was addressed by thoroughly describing participants and their context to assess transferability. Conformability was achieved through collaborative analysis and group discussions to reach a consensus on the data’s interpretation.

## Conclusion

This study indicates that several factors may have a positive influence on MWs’ performance during childbirth, including improved staffing and reduced staff rotations. HBB training could be more comprehensive, also addressing complicated airway problems that arise in daily practice and improving MW’s knowledge about neonatal physiology and the transition from fetus to the establishment of own breathing. MWs should also seek and share new information more proactively. Feelings of neglect, low appreciation and frequent societal blame contribute to decreased motivation. A well-equipped, organised hospital with ample supplies, doctor support and an effective referral system can enhance performance. Our findings can guide the development of neonatal resuscitation training in low-resource settings, though further studies are needed to identify effective, long-term strategies. It is crucial to understand why health workers perform certain procedures and to explore factors beyond immediate concerns—such as knowledge, skills, and resources—when implementing changes.

## supplementary material

10.1136/bmjopen-2024-094012 online supplemental file 1

## Data Availability

Data may be obtained from a third party and are not publicly available.
