# Supplementary material for: Midwives’ knowledge and perspectives on neonatal resuscitation and survival before and after Helping Babies Breathe training: a qualitative study in Uganda
Source: BMJ Open. 2025 Feb 7;15(2):e094012. doi: 10.1136/bmjopen-2024-094012 (PMC11808869; doi:10.1136/bmjopen-2024-094012)
Supplement: online supplemental file 1 [file bmjopen-15-2-s001.pdf]

Supplementary file

Semi-structured interview guide.

FIRST PART (40-70 minutes)

We will start with a broader discussion on midwives´ perception of their own training environment, performance, feelings, knowledge and beliefs.

1 - What is your opinion on the current HBB training at Mulago Hospital?

(Probe: How do you use the HBB skills in your clinical work? Are the skills learned by the HBB curriculum clear to you? Can you describe the key take-home messages from the HBB training? Can you describe how your clinical practice has changed after the training?)

2 - How well do you perceive that the HBB skills you have required fit within the practice setting?

(Probes: Have you had any barriers or challenges to utilising the HBB skills and equipment you received? How do you prepare before birth? How do the resuscitation guidelines look at the hospital? What happens if a child is not resuscitated according to the hospital guidelines?)

3 - How do you feel when you are about to take care of a baby with a high risk of not breathing or when a baby is not breathing?

(Probes: When do you think it is appropriate to start ventilation of a baby? Describe a good experience when resuscitating a newborn. Describe a bad experience when resuscitating a newborn.)

4 - How do you look upon ventilation, stimulation and suction of a newborn baby?

(Probes: Is it always necessary? What do you think is the purpose of ventilation? Stimulation? Suction? Do you believe there are social beliefs behind your behaviour in performing suction? If you see mucus in the mouth of a baby, according to you, can air pass down to the lungs?)

5 - Do you feel you have enough knowledge in newborn physiology?

(Probes: Describe how the lung behaves in the first minutes of life. Can air pass to the lung if there is mucus in the mouth?)

6 - According to you, how do parents and their relatives look upon babies in need of ventilation after delivery?

(Probes: Are these healthy newborns? Do you think people believe they have a good chance of survival?)

7 - According to you, how do mothers react when staff are performing neonatal resuscitation on their newborns?

(Probe: When the newborns do not start to breathe by themselves at birth? According to you, do they have the same chance of survival? Are you of the opinion that every newborn should be resuscitated?)

8 - How do you think the HBB program can be improved?

(Probes: Try to give one example of potential improvement in current training?)

Ten-minute break with tea and a snack.

SECOND PART (20-40 minutes)

We will start by showing 1-2 films illustrating representative resuscitations performed at Mulago Referral Hospital.

After each film, we will take a pause and ask:

- Please tell me your thoughts on looking at this video.

(Probes: According to you, what is good with this performance? What can be improved with this performance? Is anything about this resuscitation unclear to you? According to you, does this resuscitation adhere to the HBB guidelines?)
