# Supplementary material for: Midwives’ knowledge and perspectives on neonatal resuscitation and survival before and after Helping Babies Breathe training: a qualitative study in Uganda
Source: BMJ Open. 2025 Feb 7;15(2):e094012. doi: 10.1136/bmjopen-2024-094012 (PMC11808869; doi:10.1136/bmjopen-2024-094012)
Supplement: Supplementary file 2 [file bmjopen-2024-094012.reviewer_comments.pdf]

PEER REVIEW HISTORY

BMJ Open publishes all reviews undertaken for accepted manuscripts. Reviewers are asked to complete a checklist review form and are provided with free text boxes to elaborate on their assessment. These free text comments are reproduced below.

ARTICLE DETAILS

Title (Provisional)

Midwives' knowledge and perspectives on neonatal resuscitation and survival before and after Helping Babies Breathe training: A qualitative study in Uganda.

Authors

Myrnerts Höök, Susanna; Abrahamsson, Marielle; Namusoko, Sarah; Byamugisha , Josaphat; Bergström, Anna

---

VERSION 1 - REVIEW

Reviewer 1

Name Cavolo, Alice

Affiliation Department of Public Health and Primary Care, Centre for Biomedical Ethics and Law, KU Leuven, Leuven, Belgium

Date 05-Nov-2024

COI None

I want to sincerely thank the authors and the editor for the opportunity to read and review this excellent piece of work. The study addresses an important topic and does so with and excellent methodology, which is described in-depth in the manuscript. I highly appreciate that the authors went beyond the classic "we obtained informed consent" in the ethics considerations sections but elaborated on confidentiality and other issues. The results are clear, insightful and supported by rich and powerful quotes. The discussion is equally rich and insightful with honest and in-depth reflection on the limitations of the study. Overall, this is an excellent study and I fully endorse acceptance. I only have few small comments that, I believe, can make the result section more understandable for readers unfamiliar with the Ugandan context.

1. I commend the authors for being so concise but I think that the results section would benefit from some more elaboration, maybe providing some examples emerged from the interviews. The two points below, in particular, reu

2. P. 7 lines 26-29 "his means that MWs are frequently confronted with difficult decisions that are influenced by both professional obligations and cultural perspectives…" If possible I would like more elaboration of this statement. For example, what are some examples of

specific difficult decisions nurses are confronted with? And what are the professional obligations/cultural perspectives. How do they influence decisions?

3.. p.7-8 how does mistrust toward midwives manifest? Do people think they are not doing their job well? They do not trust their advice?

Reviewer **2**

Name **Patriksson, Katarina**

Affiliation **Department of Health Sciences, University West, Trollhättan, SE-46186, Sweden**

Date **11-Nov-2024**

COI **None**

Interesting subject but they have not do the analyze in a proper way according to Graneheim och Lundman. They have just brought the text togeher.

VERSION 1 - AUTHOR RESPONSE

Reviewer one, Dr. Alice Cavolos, comments:	We thank the reviewer for the comments.
I commend the authors for being so concise, but I think that the results section would benefit from some more elaboration, maybe providing some examples emerged from the interviews.	We have elaboration more on the result section adding more examples from the discussions and changed and added quotes. We have also restructured the result section to make it clearer. We have also changed the info in Figure 1 (previously named Table 3), to better illustrate the development of the themes.
On p. 7 lines 26-29: "this means that MWs are frequently confronted with difficult decisions that are influenced by both professional obligations and cultural perspectives…" If possible I would like more elaboration of this statement. For example, what are some examples of specific difficult decisions nurses are confronted with? And what are the professional obligations/cultural	We have further elaborated with the text explaining theme one adding info to better illustrate the difficult situations midwives are confronted with. We have added additional text and quotes. We believe this will further clarify and illustrate the midwives' working conditions.

perspectives. How do they influence decisions?	
On p.7-8:<br><br>How does mistrust toward midwives manifest? Do people think they are not doing their job well? They do not trust their advice?	We have further elaborated with the text explaining theme two adding text and quotes to further illustrate mistrust. We believe this further clarify and illustrates the situation.
Reviewer two, Dr. Katarina Patrikssons, comments:	We thank the reviewer for the comments.
Interesting subject but they have not do the analyze in a proper way according to Graneheim och Lundman. They have just brought the text together.	We acknowledge the reviewer's challenge in recognizing our adherence to the content analysis methodology outlined by Graneheim and Lundman. However, we respectfully disagree with the assertion that it was not applied, as it was consistently implemented in accordance with previous publications by the final author.<br>In response to the feedback, we have clarified the analytical steps undertaken and restructured the presentation to more explicitly demonstrate how the sub-categories, categories, and sub-themes contribute to the overarching themes. Additionally, we have revised the text, repositioned quotes to better align with their illustrative purpose, and incorporated new quotes that more effectively represent the sub-themes.<br>We have also updated Figure 1 (previously named Table 3) to improve clarity. For the sake of transparency, we have included additional information about the respondents and expanded the discussion in the "Methodological Considerations" section to further elucidate our approach to the material.<br>We hope that these revisions to the manuscript address the reviewers' concerns.
